# Elements Influencing sEMG-Based Gesture Decoding: Muscle Fatigue, Forearm Angle and Acquisition Time

**DOI:** 10.3390/s21227713

**Published:** 2021-11-19

**Authors:** Zengyu Qing, Zongxing Lu, Yingjie Cai, Jing Wang

**Affiliations:** School of Mechanical Engineering and Automation, Fuzhou University, No.2 Xueyuan Road, Fuzhou 350116, China; qingzy9264@sina.com (Z.Q.); cyj@fzu.edu.cn (Y.C.); wj_90321@sina.com (J.W.)

**Keywords:** surface electromyography, machine learning, gesture decoding, muscle fatigue, forearm angle, acquisition time

## Abstract

The surface Electromyography (sEMG) signal contains information about movement intention generated by the human brain, and it is the most intuitive and common solution to control robots, orthotics, prosthetics and rehabilitation equipment. In recent years, gesture decoding based on sEMG signals has received a lot of research attention. In this paper, the effects of muscle fatigue, forearm angle and acquisition time on the accuracy of gesture decoding were researched. Taking 11 static gestures as samples, four specific muscles (i.e., superficial flexor digitorum (SFD), flexor carpi ulnaris (FCU), extensor carpi radialis longus (ECRL) and finger extensor (FE)) were selected to sample sEMG signals. Root Mean Square (RMS), Waveform Length (WL), Zero Crossing (ZC) and Slope Sign Change (SSC) were chosen as signal eigenvalues; Linear Discriminant Analysis (LDA) and Probabilistic Neural Network (PNN) were used to construct classification models, and finally, the decoding accuracies of the classification models were obtained under different influencing elements. The experimental results showed that the decoding accuracy of the classification model decreased by an average of 7%, 10%, and 13% considering muscle fatigue, forearm angle and acquisition time, respectively. Furthermore, the acquisition time had the biggest impact on decoding accuracy, with a maximum reduction of nearly 20%.

## 1. Introduction

Over the past few decades, the field of Human Machine Interfaces (HMI) has attracted increasing interest due to its intuitive applications in the medical field. Researchers have explored signals on humans, including electroencephalography (EEG), electrocorticography (ECoG), mechanomyography (MMG) and surface electromyography (sEMG). The EEG signal has considerable practical value due to its non-invasiveness, but its signal-to-noise ratio (SNR) is low and susceptible to external interference [1]. The ECoG signal is an invasive signal, for which electrodes need to be implanted into the cerebral cortex. It has limited access to nerve information and can even cause consistent harm to the human body [2]. Compared to the previous two types of signals, MMG has the benefit of being unaffected by skin surface impedance or electrode displacement. However, its limitations include poor SNR and sensitivity to external noise [3]. Therefore, sEMG was chosen as the acquisition signal in this study.

The sEMG signal reflects the nerve activity state and is related to limb movement. During limb movement, the corresponding neural information can be obtained by back pushing sEMG signal, which has the advantages of non-invasive acquisition and bionics [4,5]. Therefore, in the past few decades, the sEMG signal has become the most popular choice for developing intuitive human-machine interfaces [6] and has been widely used in medical applications, virtual reality interfaces, nerve rehabilitation, prosthetic control, etc. [7,8,9].

The form of human muscle contraction can be divided into two categories: static and dynamic contraction. During static contraction, the length of muscle fibers does not change, and the joints do not move, but muscle fibers remain at the state of contraction [5]. Conversely, during dynamic contraction, the length of muscle fibers changes, and the joints continue to move. Therefore, the research field of motion decoding based on the sEMG signal can also be roughly divided into two categories. The first is to research the discrete motion which corresponds to static contraction of muscles through sEMG signal, such as keeping hands still or making the peace sign [10,11]. The second is to use the sEMG signal to predict the continuous motion changes of the joint which corresponds to the dynamic contraction of muscles, such as changes of joint torque and joint angle [12,13,14].

With the exploration of continuous movement still in its infancy, there is a lot of potential of advance in the future [15]. Therefore, classification accuracy improvement and prediction time reduction in gesture decoding remain the most researched issues in the sEMG signal field [16,17]. This paper explored only the decoding of discrete movements of limbs through sEMG signal. Discrete motion classification is currently the most mature and fruitful method in the field of human action decoding, based on sEMG.

The representative papers in recent years are as follows: Min et al. [18] proposed a cross individual gesture decoding method based on Long Short-Term Memory network (LSTM)—Cross individual dual network structure (CI-LSTM) in 2021. Compared with other algorithm models, the decoding accuracy of the model was improved by 9.15% on average. Wang et al. [19] used a genetic algorithm to optimize the number of signal channels and concluded that using 11 of the 16 channels can achieve 97% of the best performance of gesture decoding. Additionally, placing the electrodes in the middle of the forearm, rather than in the proximal forearm, can result in better performance. Ulysse et al. [20] applied deep learning approach to the field of gesture decoding and proposed a new migration learning scheme using convolutional neural networks, which achieved 98.31% offline decoding accuracy for 7 gestures of more than 17 participants and 68.98% offline decoding accuracy for 18 gestures of more than 10 participants. Anany et al. [12] researched the continuous decoding of forearm motion in 2019 and discovered that subject specific, hand specific and object specific decoding models offer better decoding accuracy than generic models. Additionally, Adewuyi et al. [21] analyzed the contribution of internal and external hand muscles to finger motion classification. The research showed that combining internal muscles’ sEMG data and wrist motions can significantly improve the robustness of gesture decoding.

Although the above research works have achieved promising results, most of those training and testing data were mixed in one or several days. Moreover, most of them focused on the design of appropriate channel number, feature set and classification model. However, human limb movement is a joint movement of muscles and bones controlled by the nervous system. Studies [22] have shown that different individuals have different habitual exercise patterns, and even the same person has different models of motions under different external, physical and psychological conditions. In addition, the time and frequency features of the sEMG change with the thickness and temperature of the skin, thickness of the fat between the muscle and the skin, velocity of the blood flow and location of the sEMG sensors [23]. However, the majority of the literature presented in this paper did not investigate the impact of the change of these elements on gesture decoding.

Based on this, three influencing elements were designed in this paper, namely, muscle fatigue, forearm angle and acquisition time. These elements are the most common negative factors influencing gesture decoding, based on sEMG. When the arms are held at the same position maintaining one gesture, these active tightened muscles fatigue quickly [24], and the position of the forearm is accidentally modified to induce forearm angle changes [25]. Moreover, exploring the impact of varied sEMG signal acquisition times on gesture decoding accuracy is critical for sEMG signal robustness [26].

In order to train the classification model considering these three elements, individuals were instructed to make the same gesture in different periods, different forearm angles and different muscle fatigue levels. The control variable method was then used to validate the classification accuracy of the model to compare the negative impact of these elements on gesture decoding.

The rest of this paper is organized as follows: the experimental apparatus, muscle selection, forearm angle, gesture selection and experimental settings are described in Section 2. The feature extraction and classification methods used in this experiment are reported in Section 3. The results of impact of three influencing elements for classification accuracy are discussed in Section 4. Finally, Section 5 concludes the paper and presents further research directions.

## 2. Apparatus and Experiments

The experiments were performed by five able-bodied subjects, namely, three males and two females, age = 23 ± 2, with their dominant hand. All five subjects were right hand dominant. Before the experiment, all subjects were informed about the experiment and provided the informed consent. The testing procedure was in accordance with the declaration of Helsinki.

### 2.1. Apparatus

**Figure 1:**
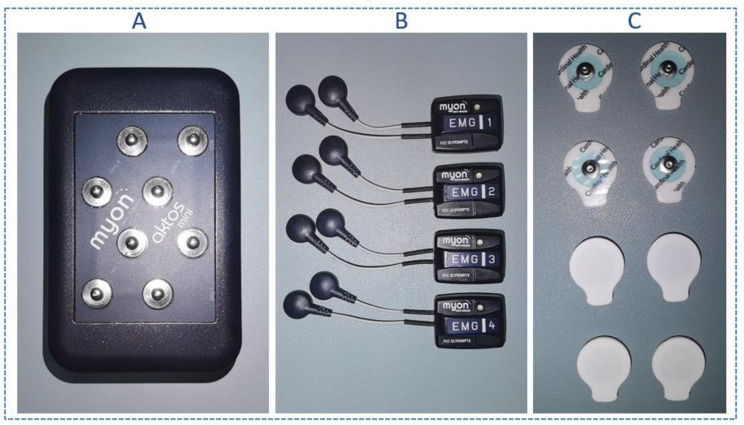
Myon Aktos-mini EMG amplifier: (**A**) signal receiving module; (**B**) signal acquisition module; (**C**) the disposable gel electrodes.

The sEMG signal of the forearm was collected by a Myon Aktos-mini EMG amplifier (Cometa company, Milan, Italy) (Figure 1). The apparatus used disposable gel electrodes (H124SG) to attach to the target muscles. The gel electrodes can provide lower skin contact impedance, reduce the influence of external interference source and improve the signal-to-noise ratio, compared to dry electrodes [4]. The sampling rate of the sEMG amplifier was 2000 Hz, and the Butterworth filter (20–500 Hz) was used for bandpass filtering.

### 2.2. Muscle Selection

**Figure 2:**
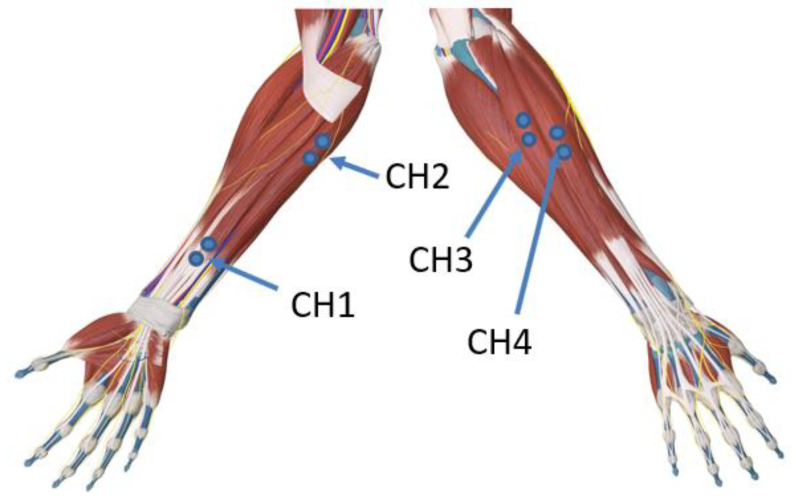
sEMG signal electrode position. Position CH1 shows the SFD; position CH2 shows the FCU; position CH3 shows the ECRL; and position CH4 shows the FE.

Since the experimental apparatus has four double electrode channels, based on previous experience and reference [27], four specific muscles were chosen for placing gel electrodes in all subjects: superficial flexor digitorum (SFD), which plays a role in finger bending except for the thumb and works in internal flexion of the wrist joint; flexor carpi ulnaris (FCU), which contracts in internal rotation of the wrist, downward wrist deviation, and elbow joint flexion; extensor carpi radialis longus (ECRL), which acts in wrist external rotation, wrist upward deviation and elbow extension; and finger extensor (FE), which works in finger extension and wrist extension except for the thumb (Figure 2).

### 2.3. Forearm Angle

**Figure 3:**
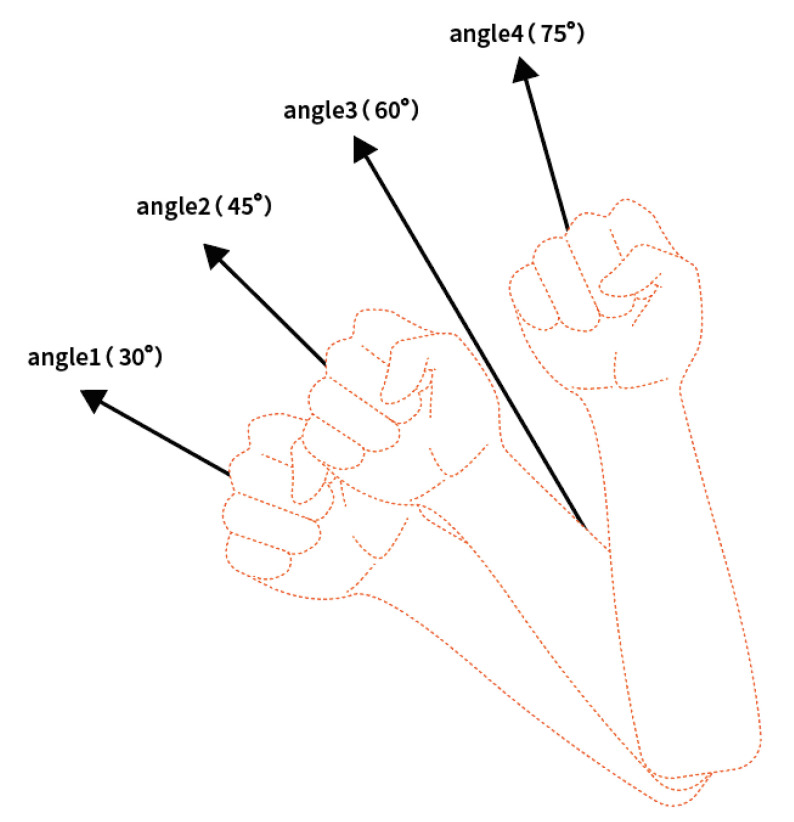
Forearm angle. Three forearm angles were selected, which were 30°, 45° and 75°.

The elbows of all subjects were placed on the table when they performed gesture movements, so the forearm angle referred to the angle between the forearm and the tabletop. In order to comprehensively analyze the negative impact of the angle on the sEMG signal from small angle difference and large angle difference, the forearm angle range and the quality of sEMG signal typically utilized in actual gesture decoding were also considered. In this paper, three forearm angles were selected, namely, 30°, 45° and 75° (Figure 3). During the experiment, the upper and lower angle deviation did not exceed ± 5°.

### 2.4. Gesture Selection

**Figure 4:**
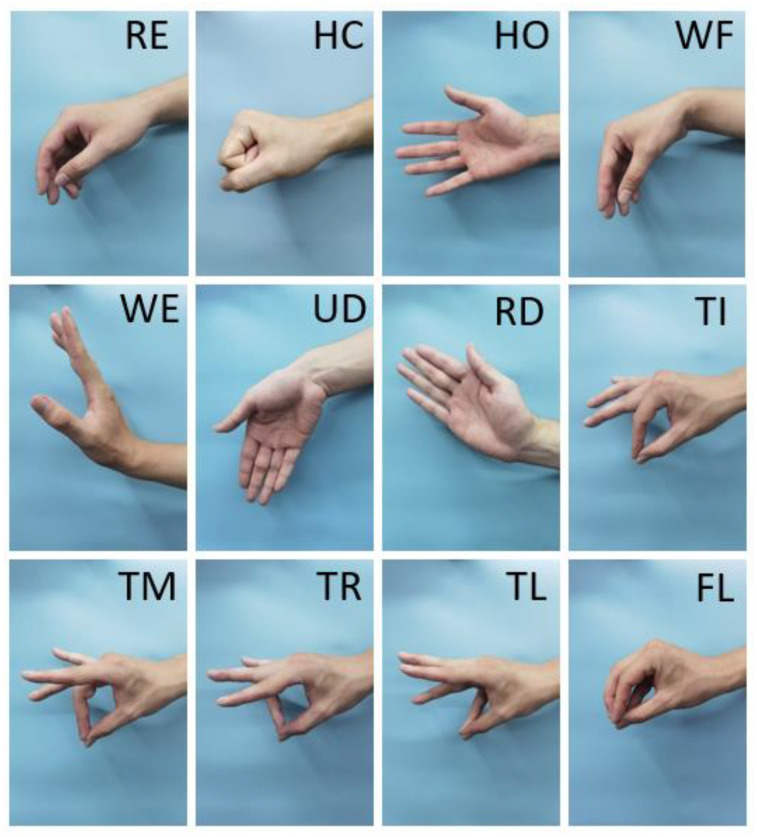
Hand movements. Three types of gestures were selected. Basic hand movements: HC and HO; wrist movements: WF, WE, UD and RD; finger movements: TI, TM, TR, TL and FL. In addition, a reference gesture RE.

In practice, hand movements can be roughly divided into three categories according to the strength and type of muscle contraction [27,28]: (1) basic hand movements: hand closing (HC) and hand opening (HO); (2) wrist movements: wrist flexion (WF), wrist extension (WE), ulnar deviation (UD) and radial deviation (RD); (3) finger movements: thumb touches index finger (TI), middle finger (TM), ring finger (TR), little finger (TL), and the five fingertips touch (FL) (Figure 4). These hand movements basically cover the common gestures in daily life. In addition, there was a relax gesture (RE) as a reference, which was not analyzed.

### 2.5. Experimental Setting

Each subject was provided with a motion instruction regarding how the experimental task was to be performed. For all experiments, subjects sat up straight, put their elbow on the table, exerted slight force on their arms, and tried to retain the force of each hand movement as consistent as possible.

The sEMG signal was sampled for five consecutive days, with an interval of one day for each sampling. Therefore, the signal data were measured three times totally, and were divided into categories A, B and C. Forearm angles were also divided into 30°, 45°, and 75° respectively. Starting from the relaxation gesture, each gesture lasted for 5 s, and the interval between each gesture was 10 s. A group of 11 gestures was made up the normal muscle group and recorded as Class a; then, restarting in a short time from the relaxation gesture, each gesture lasted for 15 s, and there was no interval between each gesture. A group of 11 gestures was made up the fatigue muscle group, regarded as Class b.

Regarding muscle fatigue, it has been demonstrated that as muscle fatigue continues, the frequency domain power spectrum shifts to the low frequency direction [24]. At the same time, the time domain feature RMS will increase, and Mean Power Frequency (MPF) feature of the frequency domain will decrease [29,30]. As a result, these two eigenvalues have been frequently used as sensitive muscle tiredness indicators.

**Figure 5:**
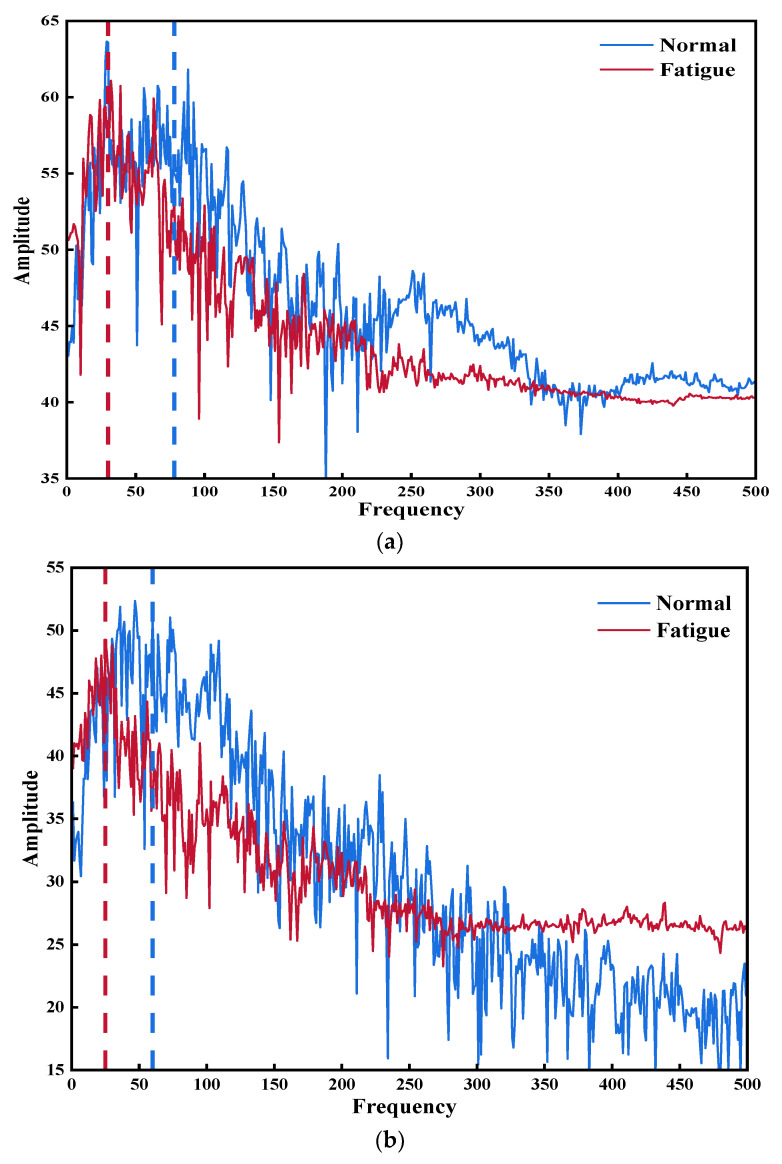

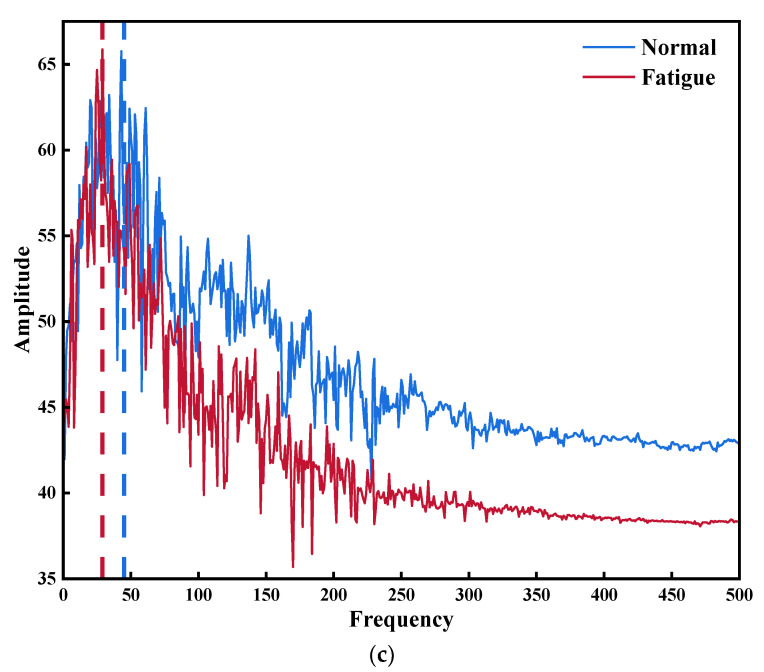
The Power spectrums of three selected hand gestures: (**a**) power spectrum of HO; (**b**) power spectrum of UD; (**c**) power spectrum of TM.

In this paper, all subjects were performed the muscle fatigue verification experiment at a 45° forearm angle according to the experimental description above; then, the power spectrum of the sEMG signal was analyzed, as shown in Figure 5. Due to paper space limitations, one of each type of gestures of one subject was chosen as the representation, namely, HO, UD and TM. Additionally, the RMS and MPF features of each gesture of same subject were analyzed (Table 1). The results show that the spectrum of fatigued muscle group (Class b) goes to the left when compared to the spectrum of normal muscle group (Class a), indicating that the power spectrum shifts to the low frequency during muscular exhaustion. Moreover, the RMS and FPM values in Table 1 increased and decreased, respectively, indicating the presence of muscular exhaustion.

It must also be noted that due to factors such as gesture delay and error, when extracting valid gesture data, the first second and last second of each sampling were discarded, and only the middle three seconds were retained as valid data in Class a, and only 12–14 s were retained as valid data in Class b.

In order to avoid muscle accumulated fatigue, the relaxation time after each Class a and Class b was ten minutes. Taking into account three different issues, 18 types of datasets could be obtained, and each type of dataset had five groups. The detailed dataset classification is shown in Table 2.

## 3. Methodology

The raw signal was collected to obtain the dataset, and then, segmentation was needed. A shorter window size increases the time resolution and captures more complex gestures. However, a window size that is too small might lead to classification error. On the contrary, longer window length can be used to avoid high deviation and variance but will cause user perception delay [17,31]. In this paper, a 400 ms window size with 50 ms increments was used to split the raw sEMG signal.

### 3.1. Feature Extraction

Generally, the most important factor for reliable classification results for any signal is the extraction of discriminating features [32]. The extracted features for sEMG signal analysis can be divided into three categories, namely, Time Domain (TD), Frequency Domain (FD) and Time-Frequency Domain (TFD) features [33]. TD features contain the information about the sEMG signal that is extracted from signal amplitude, while FD features contain information regarding the power spectral density of the signal. TFD features can characterize varying frequency information at different time locations, providing non-stationary information of the analyzed signal. Compared with the other two types of features, because of their low computational difficulty, small amount of computation and good classification performance, time-domain features are widely used [34,35].

In this paper, four TD features were used to extract the sEMG signal, namely Root Mean Square (RMS), Waveform Length (WL), Zero Crossing (ZC) and Slope Sign Change (SSC) [36,37]. Each channel had four features, and each apparatus had four channels. Therefore, the input dimensions of classification were sixteen, and because there were fewer input dimensions, no dimensionality reduction but normalization was required.

(1) Root Mean Square: RMS is the square root of the average power of the signal at a given time. This feature quantifies the effort of the muscle. RMS is defined as follows:
  (1) $$RMS=\sqrt{\frac{1}{N}\sum_{i=1}^{N}{\left(x_{i}\right)}^{2}}\;\;\;,\;\;$$
  where N is the length of the window size and i is the ith sample point.
(2) Waveform Length: WL is the measurement of the waveform amplitude, frequency and duration of the signal, which is an index to measure the complexity of the signal. WL is defined as follows:
  (2) $$WL=\sum_{i=2}^{N}\left|x_{i}-x_{i-1}\right|,\;\;\;$$
  where N is the length of the window size and i is the ith sample point.
(3) Zero Crossing: ZC is the number of zero crossing at a given time period. ZC provides important information regarding the FD characteristics of the signal and is an important indicator of muscle fatigue. ZC is defined as follows:
  (3) $$ZC=\sum_{i=2}^{N}(-x_{i}x_{i-1}>0\;\;\&\&\;\;\left|x_{i}-x_{i-1}\right|>\varepsilon ),\;$$
  where N is the length of window size, i is the ith sample point and ε is the voltage threshold, which was selected referring to the signal noise of the skin surface. In this paper, the threshold was set at 20 mV.
(4) Slope Sign Change: SSC is the number of times the slope of the measured waveform changes signs. It provides important information about the FD characteristics and is defined as follows:
  (4) $$SSC=\sum_{i=2}^{N-1}(\Delta x_{i+1}\Delta x_{i-1}>0\;\;\&\&\left(\left|x_{i+1}|>\varepsilon \;\;or\;\;|x_{i-1}\right|>\varepsilon \right)),\;$$
  where N is the length of the window size, i is the ith sample point, and ε is the voltage threshold, which was selected referring to the signal noise of the skin surface. In this paper, the threshold was set at 20 mV.

### 3.2. Classification Model

As the last step of gesture decoding, selecting an appropriate classification model will help improve the recognition accuracy and generalization ability. At present, commonly used classification models include Linear Discriminant Analysis (LDA), K-Nearest Neighbor (KNN), Bayes, Support Vector Machine (SVM), Neural Network, etc. In this paper, LDA and Probabilistic Neural Network (PNN) were selected as the classification models.

#### 3.2.1. LDA Classification Model

**Figure 6:**
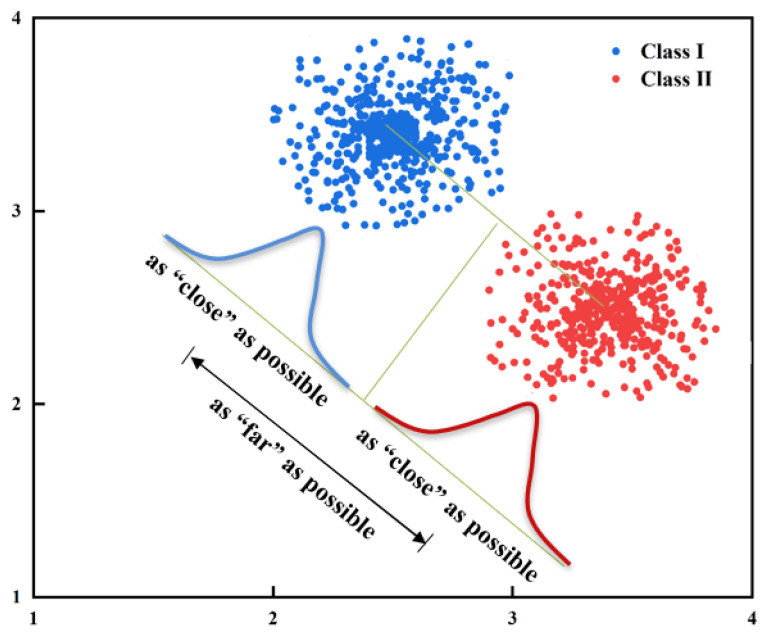
LDA classification model diagram.

LDA classification learning is a classic linear learning algorithm. Its principle is simple: Given a training dataset, try to project the dataset into a low-dimensional space, making the projection points of the same data as close as possible while the projection points of different data as far as possible (Figure 6). The data from the testing datasets are projected into the same low dimensional space, and then classified according to the position of the projection points.

On one hand, to make the projection points of same data lie close to one another, their covariance should be minimized. On the other hand, the distances between centers of different kinds of classes need to be far away causing the projection spacing of them to be larger. Here, two concepts are introduced. The within-class scatter matrix is defined as

(5) $$S_{\omega }=\sum_{i=1}^{N}\sum_{x\epsilon X_{i}}\left(x-\mu_{i}\right){\left(x-\mu_{i}\right)}^{T},\;\;$$

where N is the number of classes; $X_{i}$ and $\mu_{i}$ are the set and mean vector of the ith class, respectively. The inter-class scatter matrix is defined as

(6) $$S_{b}=\sum_{i=1}^{N}m_{i}\left(\mu_{i}-\mu \right){\left(\mu_{i}-\mu \right)}^{T},$$

where $m_{i}$ is the number of ith class, and μ is the mean vector for all classes. Considering both intra-class and inter-class scatter matrices, we can achieve classification through Formula (7):

(7) $$\underset{W}{\max}\frac{tr\left(W^{T}S_{b}W\right)}{tr\left(W^{T}S_{\omega }W\right)}\;,\;\;\;$$

where the closed-form solution of matrix $W\in R^{d\times \left(N-1\right)}$ is composed of $d^{\prime }$ largest non-zero generalized eigenvalues corresponding to the eigenvectors of $S_{\omega }^{-1}S_{b}$; d and $d^{\prime }$ are the dimensions of features before and after projection; $tr\left(\cdot \right)$ is the trace of the matrix.

#### 3.2.2. PNN Classification Model

**Figure 7:**
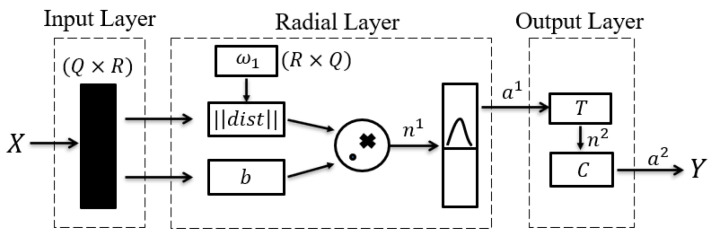
PNN classification model diagram.

Probabilistic Neural Network (PNN) was first proposed by Dr. D.F. Specht in 1989. Currently, it is commonly used for pattern classification. Its schematic diagram is shown in Figure 7:

The weight of radial layer $\omega_{1}$ is directly taken from input dataset X, and dimension Q of X corresponds to the number of neurons in the radial layer. When X is input into the network, the distance between X and $\omega_{1}$ is obtained using the Euclidean distance and stored in *dist*. The value in *dist* is then multiplied by the polarization factor b point by point. The results are applied to the radial basis function provided as output $a^{1}$.

It is worth noting that when polarization factor b is too small, the input of activation function $n^{1}$ will become smaller; therefore, output $a^{1}$ will increase following radial basis function conversion, which will improve the contribution of the training dataset to the current dataset, leading data overfitting. On the contrary, if polarization factor b is too large, the contribution of the training dataset to the current dataset will decrease, leading to data underfitting.

The weight of the competition layer was set as the expected value vector matrix T, in which each row vector had only one element of 1, representing the corresponding category; the remaining elements were 0, and then, the product of the matrix T and the radial layer output $a^{1}$ was calculated. Finally, $n^{2}$ was obtained through the competition calculation of the transfer function of the competition layer, the larger element took the value 1, and the rest was 0, so that the classification of the input vector could be completed.

As a variant of the RBF network, PNN network has a simple structure and few parameters to be adjusted. Furthermore, it can achieve arbitrary nonlinear approximation. Compared with the traditional BP network, the training time of the PNN network is only slightly longer than the time of reading data and has global convergence, which is suitable for real time gesture decoding.

### 3.3. Learning Framework

**Figure 8:**
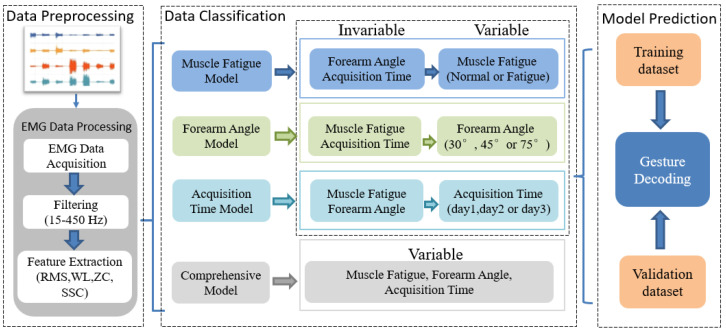
Block diagram of proposed learning framework.

In this paper, the influences of the subjects at different muscle fatigue levels, forearm angles and acquisition times on gesture decoding were studied. To this end, the TD features from sEMG data were extracted. A detailed flow is described in Figure 8. The features datasets were then divided into two sets, one for training and the other for decoding. To control variables, the four different categories were divided for analysis, namely:

(1) Influence of Muscle Fatigue (MF): Compare the effects of different muscle fatigue levels (normal or fatigue), same acquisition time and forearm angle on gesture decoding accuracy.
(2) Influence of Forearm Angle (FA): Compare the effects of different forearm angles (30°, 45°, 75°), same muscle fatigue level and acquisition times on gesture decoding accuracy.
(3) Influence of Acquisition Time (AT): Compare the effects of different acquisition times (day1, day2,day3), same muscle fatigue level and forearm angles on gesture decoding accuracy.
(4) Influence of MF, FA and AT: Compare the effects of different muscle fatigue, forearm angle and acquisition time on gesture decoding accuracy.

## 4. Results and Discussions

In the practical application of gesture decoding based on the sEMG signal, trained classification models have never used a validation dataset. In order to fit the accuracy of the actual classification model for gesture decoding, the commonly used cross validation method (dividing the dataset into k mutually exclusive subsets of similar size, then successively selecting the union of k-1 subsets as the training set and the remaining subsets as the test set) was not employed; instead, the method in which the training and testing datasets are completely separated from each other was used (data from the testing dataset were not used for training). Furthermore, the results were the mean values obtained by disrupting the data cycle training ten times. Additionally, decoding accuracy was selected as validation metric.

### 4.1. Influence of Muscle Fatigue

**Figure 9:**
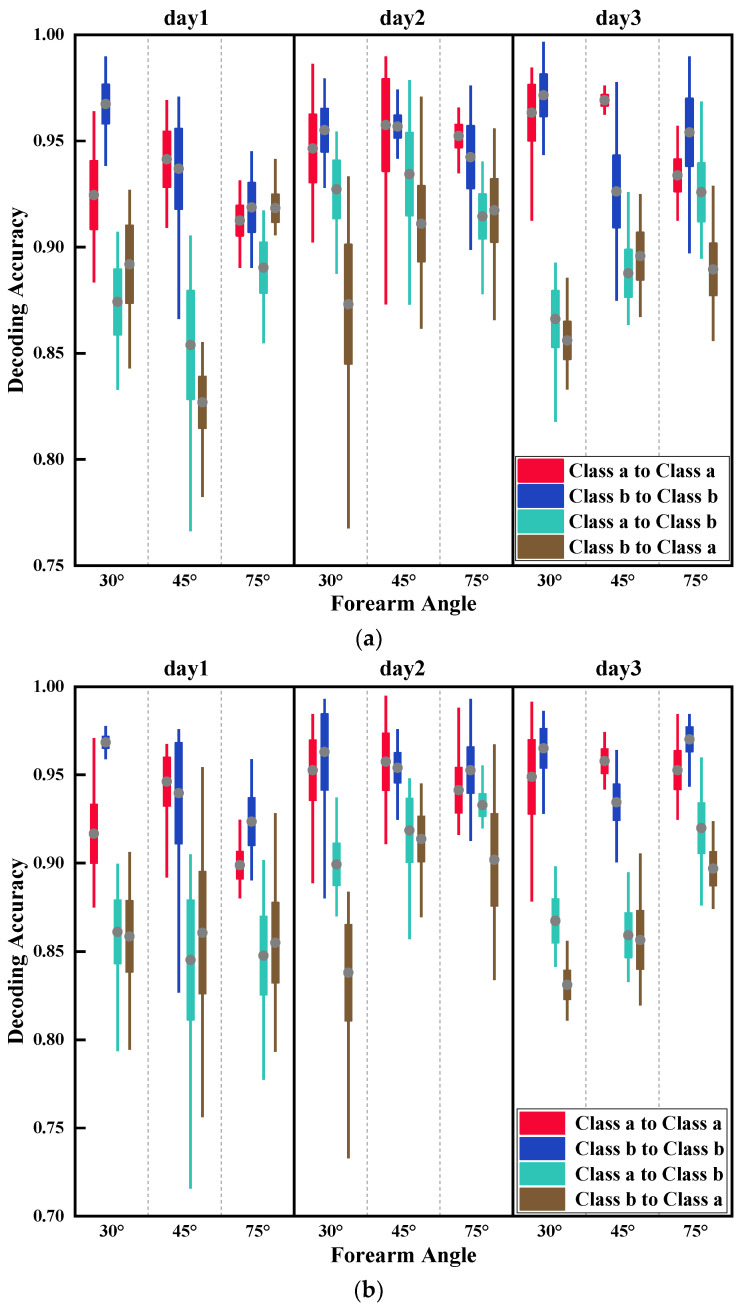
Influence of muscle fatigue on the accuracy of gesture decoding. Class a to Class a means to use Class a datasets as training and testing datasets, and the same goes for Class b to Class b, while Class a to Class b means to use Class a datasets as training datasets, Class b as testing datasets, and the same goes for Class b to Class a. (**a**) Influence of muscle fatigue using LDA; (**b**) influence of muscle fatigue using PNN.

In this section, Class a datasets trained models were used to test Class a and Class b datasets, respectively, to obtain the effect of muscle fatigue on the accuracy of gesture decoding. At the same time, Class b datasets trained models were used to test Class a and Class b datasets, respectively. Results are shown in Figure 9.

The results obtained by the two models using Class a and Class b as both training and testing datasets, respectively, were close, and average decoding accuracies were approximately 95%. When using Class a to test Class b and Class b to test Class a, the results obtained by the two models were also close. However, compared to the first two types of models, a considerable reduction was observed in the average decoding accuracies, which were close to 88%. In addition, decoding accuracy rates fluctuated greatly, with a maximum gap of nearly 15%. This pointed to the effect muscle fatigue has on the accuracy of gesture decoding, making it unstable. Additionally, a Pearson correlation analysis was performed on each subject to verify the universality of the influence of muscle fatigue on the model (Table 3). When compared to using Class a datasets for both training and testing, using Class b datasets for both training and testing datasets instead resulted in lower correlation coefficient R values. This could be related to the fact that muscle fatigue in each participant was unpredictable. Nevertheless, almost all R values were higher than 0.4. Most of the R values of Class a to Class b and Class b to Class a in each subject were over 0.5.

### 4.2. Influence of Forearm Angle

The results from the previous section showed that muscle fatigue had an impact on the accuracy of gesture decoding. Furthermore, under same conditions, when using Class a and Class b as training and testing datasets, respectively, while using Class a to test Class b and Class b to test Class a, the results were close. Therefore, it is reasonable to assume that when comparing the effect of forearm angle and acquisition time on the classification models, the accuracies of using single Class a or Class b datasets will be similar. Here, only Class a datasets were used in this section, for paper limitation reasons.

**Figure 10:**
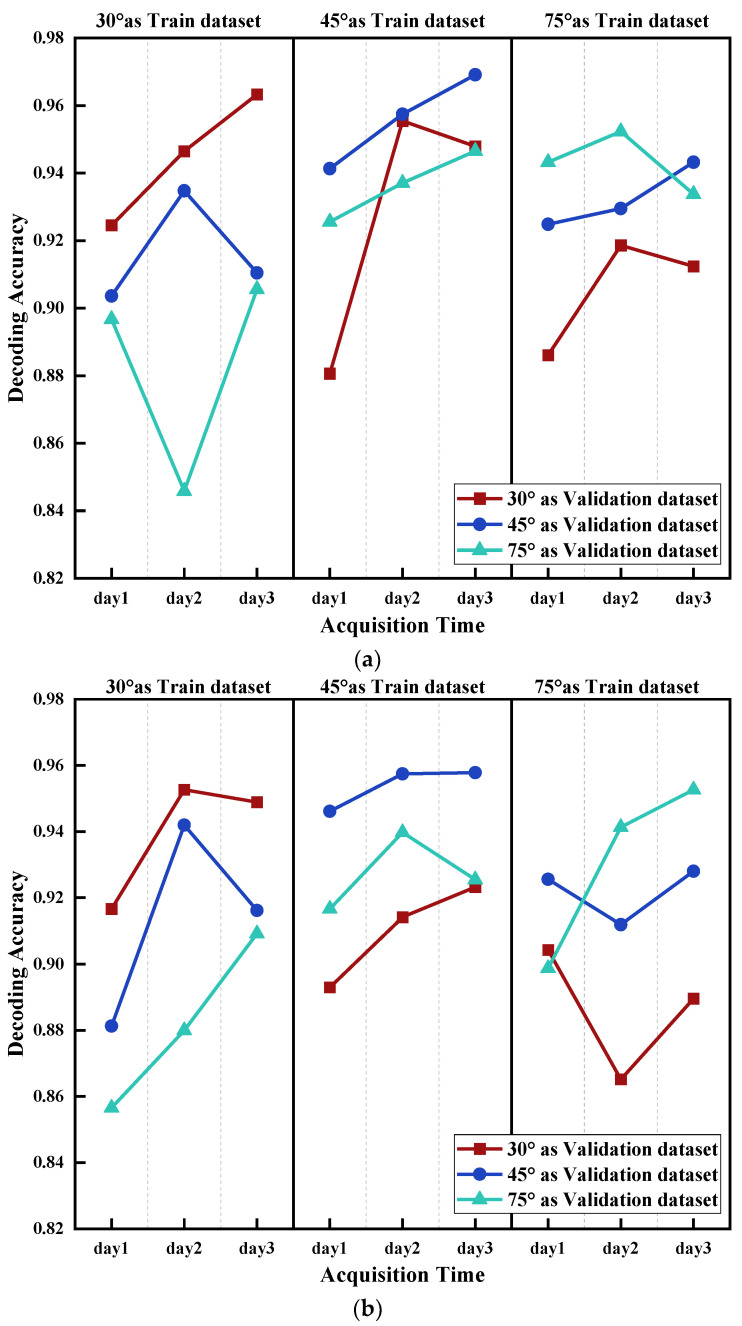
Influence of forearm angle on the accuracy of gesture decoding. 30° as validation dataset, 45° as Validation dataset and 75° as Validation dataset means using Class 30°, Class 45° and Class 75° to test the trained model, respectively. (**a**) Influence of forearm angle using LDA; (**b**) Influence of forearm angle using PNN.

To avoid the influence of signal acquisition time, the training datasets and corresponding testing datasets were collected on the same day. The datasets with forearm angles of 30°, 45° and 75° collected in three days were used to test the datasets with forearm angles of 30°, 45° and 75° collected on the same day. The results are shown in Figure 10.

The results of the above two classification models showed that when using same angle data for both training and testing, the accuracies of the two models were the highest. However, the greater the gap of forearm angle was the lower the decoding accuracy. For instance, when 30° of forearm angle was used as training dataset and 45° and 75° as testing datasets, the average decoding accuracies decreased by about 3% and 7%, respectively, compared to using 30° as testing dataset. At the same time, when the forearm angle of 45° was used as training dataset to test 30° and 75°, the decoding accuracies returned by the classification models were similar. It is confirmed that forearm angle had an impact on the accuracy of gesture decoding, and the greater the angle difference, the bigger the impact was. Moreover, a Pearson correlation analysis was performed on each subject to verify the universality of the influence of forearm angle on the model (Table 4). It can be seen that almost all R values were greater than 0.7 except for subject 1 in 45 as validation dataset, confirming the universality of the impact of forearm angle.

### 4.3. Influence of Acquisition Time

**Figure 11:**
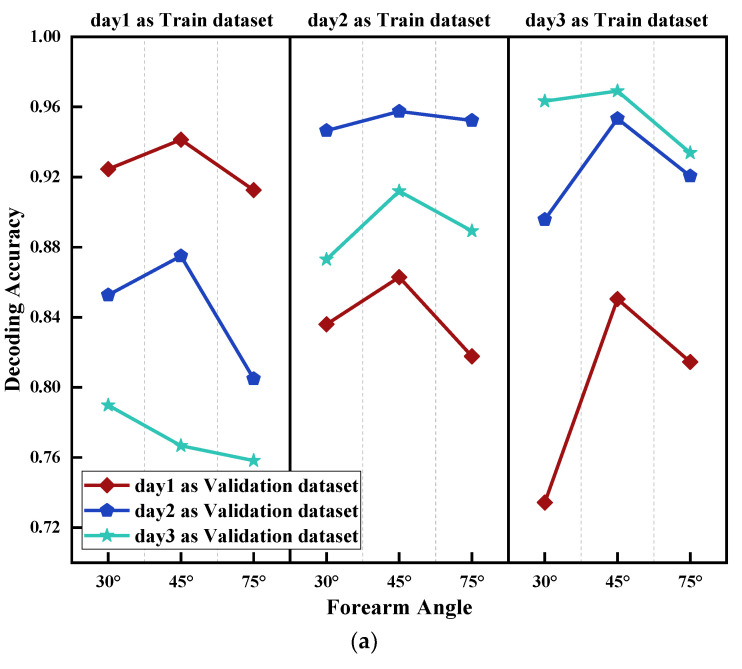

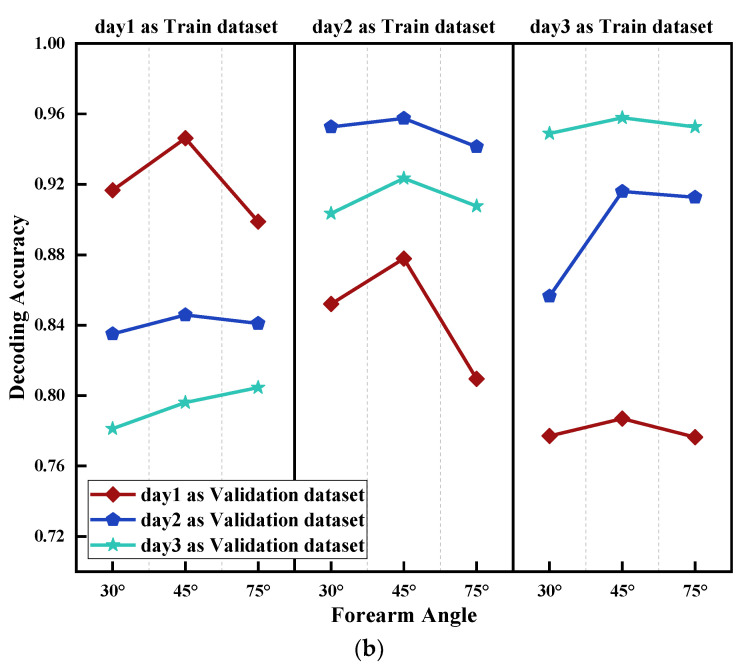
Influence of acquisition time on the accuracy of gesture decoding; day1 as Validation dataset, day2 as Validation dataset and day3 as Validation dataset means using Class day1, day2 and day3 to test trained model, respectively. (**a**) Influence of acquisition time using LDA; (**b**) Influence of acquisition time using PNN.

Similar to Section 4.2, the impact of acquisition time on gesture decoding was discussed in this section. In order to control the impact of muscle fatigue and forearm angle, only Class a datasets were used. Additionally, datasets with acquisition time of day1, day2,day3 were used to test the datasets of acquisition time of day1, day2,day3, respectively, with the same forearm angle (Figure 11).

From the results in Figure 11, it became clear that when using the same acquisition time data as both training and testing datasets, the accuracies of the two models were the highest, and the average decoding accuracies were close to 95%, which were similar to the results of forearm angle. Moreover, the bigger the gap of acquisition time was, the lower the decoding accuracy. For example, using day1 as training dataset, day3 as testing dataset, while using dataset day3 to test day1, the average decoding accuracies both decreased by more than 20%. Nevertheless, models trained by day2 were used to test datasets day1 and day3 respectively, and average decoding accuracies decreased lower than the above models, by approximately 5–10%. Similarly, to the previous section, Pearson correlation coefficient was used to validate the universality of the conclusion (Table 5). The majority of R values were rarely lower than 0.95. This is due to the fact that acquisition time had a considerable impact on gesture decoding (maximum above 20%), and the longer the collection time span, the more severe the impact. The Pearson correlation coefficient became insensitive in this instance, and tiny variations in the decoding accuracy of various participants will not induce changes in the correlation coefficient. Additionally, all subjects showed the same trend.

In summary, among factors such as muscle fatigue, forearm angle and acquisition time, the acquisition time had the greatest impact on the accuracy of gesture decoding, which may also be related to the position offset of the disposable electrode patch at different acquisition times.

### 4.4. Influence of MF, FA and AT

The three influencing factors (i.e., different muscle fatigue level, different forearm angle and different acquisition time) were included in each classification model. Therefore, 18 classification models were designed within three categories (AB_C, BC_A, CA_B) for each subject (Table 6).

**Figure 12:**
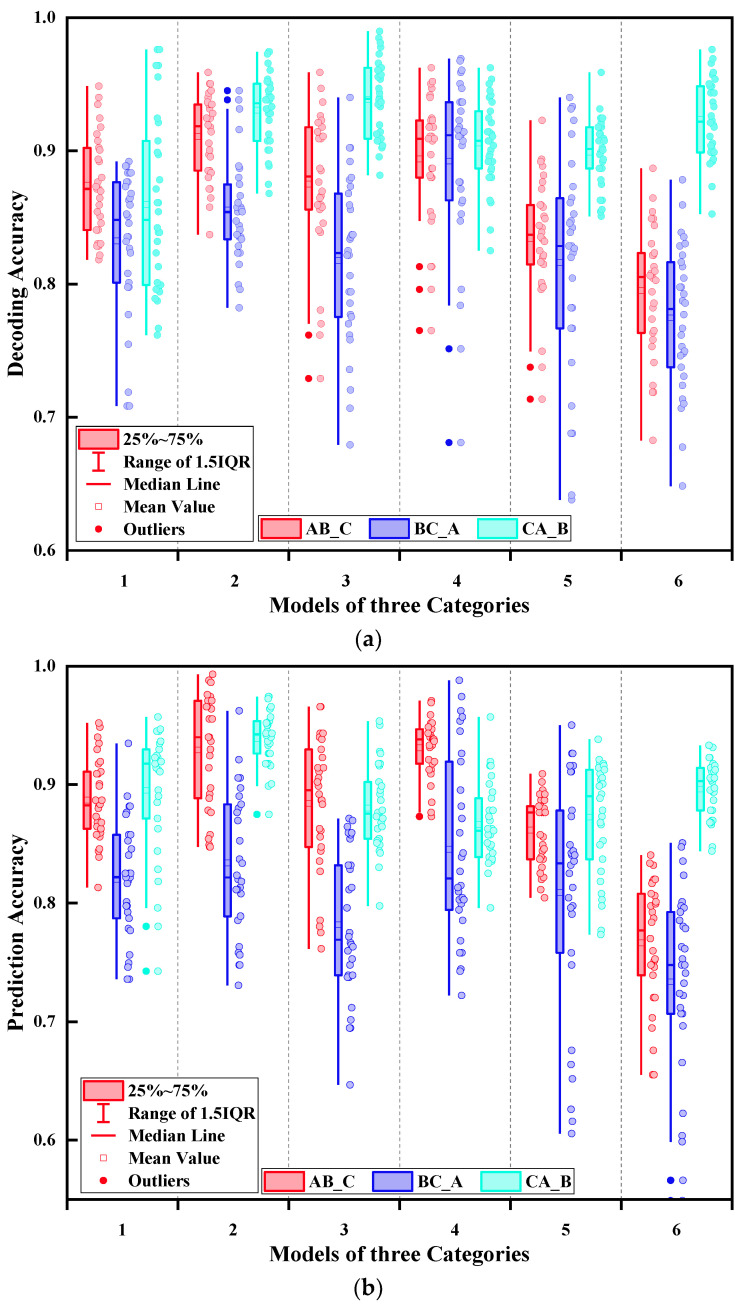
Influence of MF, FA and AT on the accuracy of gesture decoding. AB_C means using Class day1 and day2 as training datasets and using Class day3 as testing dataset. The same applies for BC_A and CA_B. (**a**) Influence of MF, FA and AT using LDA; (**b**) influence of MF, FA and AT using PNN.

For example, in AB_C category, the data collected by day1 and day2 were used for training, and data acquired by day3 were used for testing. For the first model in AB_C category, two of the five groups in A_b_30 and B_a_45 were randomly selected, respectively, to form training datasets and then choose one of five groups successively in C_a_75 as testing dataset, and the same procedures were followed for the other 17 models. To lower bias in the experimental results, the participants with the best and worst decoding performance were eliminated, and then, ten decoding result points were deducted from each of the three subjects in the center, for a total of 30 points in each model. The obtained results are shown in Figure 12.

According to the results, the decoding accuracy rates of all major categories were not stable, with a maximum rate gap of more than 25%. Conversely, it was easy to find that the trend of decoding accuracies between AB_C and BC_A was similar, but the average accuracies of AB_C were higher than that of BC_A, nearly 5–10%. On the other hand, the trend of decoding accuracies of CA_B was stable, which was different from the other two categories. As a result, it is postulated that a major reason for this situation was the different acquisition time. Moreover, when choosing bigger gap of forearm angle data as training datasets (randomly choose two out of five groups in angle of 30° and 75°, respectively), the average decoding accuracies generally deteriorated.

## 5. Conclusions

In this paper, the effects of muscle fatigue, forearm angle and acquisition time on the validation accuracy of gesture decoding were investigated. For this purpose, four specific muscles (i.e., SFD, FCU, ECRL and FE) and 11 hand movements, commonly used in daily life, were selected. Meanwhile, four TD features (RMS, WL, ZC, and SSC) and two classification models (LDA and PNN) were chosen to analyze the sEMG signal.

The analysis of the signal was performed in four parts: The first part was an analysis of the influence of muscle fatigue on the accuracy of gesture decoding. The second and third parts were analyses of the influence of forearm angle and acquisition time on the accuracy of gesture decoding. The final part was a comprehensive analysis of the effect of the three elements mentioned above on gesture decoding accuracy.

From Section 4.1, it was concluded that muscle fatigue had an impact on the classification model for gesture decoding, by decreasing the average accuracy by approximately 7%. Nevertheless, the validation accuracy exceeded 88%, which showed that the negative impact of muscle fatigue was relatively small (Figure 9). Based on the results of Section 4.2 and Section 4.3, it can be concluded that when using same forearm angle or acquisition time data as both training and testing datasets, the decoding accuracies of the two models were the highest, both close to 95%. Furthermore, it was observed that the average test accuracy was deteriorating with the increasing gap of forearm angle and acquisition time. However, the negative impact on the accuracy of gesture decoding of acquisition time was substantially higher than that of forearm angle. The maximum accuracy decrease of acquisition time was more than 20%, whereas that of forearm angle was less than 10%. Finally, the impact of three influencing elements on the classification model was comprehensively considered. The results showed that the decoding accuracy of each category was not stable. Among them, AB_C and BC_A had a similar accuracy trend, and the average accuracies of AB_C were higher than that of BC_A, close to 5–10%. However, the trend of average decoding accuracies of CA_B was relatively stable.

On the one hand, human hand movement is one of the most complex limb movements, with many factors that hinder gesture decoding. On the other hand, as a non-invasive signal connected to limb movement, sEMG has several advantages in an intuitive human–computer interface, including convenience, freedom of space and light constraints. Therefore, the study of sEMG-based gesture decoding opens new opportunities for future practical applications, such as prosthetic control and virtual reality.

Although this article has provided theoretical and experimental results, many issues that need further discussion still remain. First, the conditions for classifying muscle fatigue levels are only outlined, so a more precise and effective definition of the conditions for muscle fatigue classification is needed. Second, this paper only explored the EMG signals of the three angles of the forearm when the human body sits upright, which only covers a small part of the actual human movement. Therefore, more scenarios need to be explored. Third, this article only used the EMG data collected in three days. In the future, we will research the changes of EMG signal over time and the impact on the classification model of gesture decoding in a longer time span. Finally, the experiment described in this paper was carried out in a university laboratory, and the volunteers there were graduate students pursuing a master’s degree, so they were young and had little age difference. Furthermore, the problem of the left-hand or right-hand dominant was not considered in this research. In the following expansion experiment, volunteers of various ages and dominant hands will be recruited to carry out the experiment in order to ensure that the experimental outcomes are universal.

## Figures and Tables

**Table 1:** Comparison between muscle normal and fatigue of each gesture using RMS and FPM.

Gesture	RMS		FPM	
	Normal	Fatigue	Normal	Fatigue
HC	182.23	252.05	90.35	65.45
HO	168.33	227.97	95.36	82.63
WF	71.61	118.07	132.28	65.70
WE	87.10	118.75	137.46	105.80
UD	116.77	141.20	94.69	76.93
RD	200.10	229.87	119.02	103.70
TI	80.76	109.14	124.95	75.34
TM	138.16	172.96	84.46	59.37
TR	111.89	104.92	76.07	51.34
TL	154.70	132.99	99.48	94.76
FL	138.62	108.16	86.60	98.89

**Table 2:** sEMG signal datasets classification.

		FA (30°)	FA (45°)	FA (75°)
Day1	Normal	A_a_30	A_a_45	A_a_75
	Fatigue	A_b_30	A_b_45	A_b_75
Day2	Normal	B_a_30	B_a_45	B_a_75
	Fatigue	B_b_30	B_b_45	B_b_75
Day3	Normal	C_a_30	C_a_45	C_a_75
	Fatigue	C_b_30	C_b_45	C_b_75

**Table 3:** Correlation analysis on each subject for the influence of muscle fatigue.

	Model	Subj.1	Subj.2	Subj.3	Subj.4	Subj.5
Class a to Class a	LDA	0.6409	0.8109	0.7111	0.4123	0.5616
	PNN	0.4987	0.6057	0.6943	0.5313	0.5413
Class b to Class b	LDA	0.4884	0.8545	0.3950	0.6125	0.4910
	PNN	0.4402	0.6866	0.4828	0.4836	0.5880
Class a to Class b	LDA	0.5360	0.9183	0.6695	0.6302	0.6870
	PNN	0.6141	0.7128	0.7331	0.7391	0.6751
Class b to Class a	LDA	0.9255	0.5556	0.5789	0.5843	0.7746
	PNN	0.3734	0.5210	0.6684	0.6731	0.6448

**Table 4:** Correlation analysis on each subject for the influence of forearm angle.

Trend	Model	Subj.1	Subj.2	Subj.3	Subj.4	Subj.5
30° as Validation dataset	LDA	0.8437	0.8741	0.7607	0.7948	0.8622
	PNN	0.8273	0.8542	0.8206	0.7484	0.9582
45° as Validation dataset	LDA	0.5925	0.9974	0.9486	0.9368	0.7738
	PNN	0.6883	0.8715	0.9388	0.9458	0.8211
75° as Validation dataset	LDA	0.9536	0.9428	0.9797	0.7718	0.9981
	PNN	0.9147	0.9358	0.9486	0.7408	0.9429

**Table 5:** Correlation analysis on each subject for the influence of acquisition time.

Trend	Model	Subj.1	Subj.2	Subj.3	Subj.4	Subj.5
day1 as Validation dataset	LDA	0.9881	0.9995	0.9929	0.9815	0.9865
	PNN	0.9916	0.9996	0.9736	0.9697	0.9938
day2 as Validation dataset	LDA	0.9884	0.9837	0.9581	0.9772	0.9620
	PNN	0.8750	0.9627	0.9940	0.9812	0.9913
day3 as Validation dataset	LDA	0.9960	0.9987	0.9289	0.9996	0.9984
	PNN	0.9941	0.9960	0.9515	0.9907	0.9869

**Table 6:** Classification of sEMG model.

**Model**	**AB_C (6 Models)**		
1	A_b_30	B_a_45	C_a_75
2	A_a_30	B_b_45	C_b_75
3	A_a_45	B_b_75	C_a_30
4	A_b_45	B_a_75	C_b_30
5	A_a_75	B_a_30	C_b_45
6	A_b_75	B_b_30	C_a_45
**Model**	**BC_A (6 Models)**		
1	B_b_30	C_a_45	A_a_75
2	B_a_30	C_b_45	A_b_75
3	B_a_45	C_b_75	A_a_30
4	B_b_45	C_a_75	A_b_30
5	B_a_75	C_a_30	A_b_45
6	B_b_75	C_b_30	A_a_45
**Model**	**CA_B (6 Models)**		
1	C_b_30	A_a_45	B_a_75
2	C_a_30	A_b_45	B_b_75
3	C_a_45	A_b_75	B_a_30
4	C_b_45	A_a_75	B_b_30
5	C_a_75	A_b_30	B_b_45
6	C_b_75	A_b_30	B_a_45

## Data Availability

Data sharing is not applicable to this article. Please contact the authors for further requests.
